# Circulating and Adipose Tissue mRNA Levels of Zinc-α2-Glycoprotein, Leptin, High-Molecular-Weight Adiponectin, and Tumor Necrosis Factor-Alpha in Colorectal Cancer Patients With or Without Obesity

**DOI:** 10.3389/fendo.2018.00190

**Published:** 2018-04-26

**Authors:** Huijuan Zhu, Meijuan Liu, Nianrong Zhang, Hui Pan, Guole Lin, Naishi Li, Linjie Wang, Hongbo Yang, Kemin Yan, Fengying Gong

**Affiliations:** ^1^Key Laboratory of Endocrinology of National Health and Family Planning Commission, Department of Endocrinology, Peking Union Medical College Hospital, Chinese Academy of Medical Science, Peking Union Medical College, Beijing, China; ^2^Department of General Surgery, Peking Union Medical College Hospital, Chinese Academy of Medical Science, Peking Union Medical College, Beijing, China

**Keywords:** colorectal cancer, adipokines, zinc-α2-glycoprotein, obesity, leptin

## Abstract

**Objectives:**

To explore zinc-α2-glycoprotein (ZAG), leptin, high-molecular-weight adiponectin (HMW-ADPN), and tumor necrosis factor-alpha (TNF-α) levels in serum and subcutaneous and visceral white adipose tissue (sWAT and vWAT) among normal weight (NW) and overweight/obese (OW/OB) patients with colorectal cancer (CRC).

**Methods:**

A total of 76 Chinese CRC patients (42 NW + CRC, 34 OW/OB + CRC) and 40 healthy controls were recruited. Serum levels of the adipokines of interest were measured by an enzyme-linked immunosorbent assay method, and their mRNA levels in sWAT and vWAT were determined by reverse transcription quantitative PCR methods.

**Results:**

Serum ZAG levels in the NW + CRC group were significantly increased by 11.7% compared with the healthy controls. Serum leptin levels in the OW/OB + CRC group were found to be increased by 57.7%, while HMW-ADPN levels were decreased by 23.5% when compared with the NW + CRC group of CRC patients. Additionally, *ZAG* mRNA levels in sWAT were significantly reduced by 78.8% in OB + CRC in comparison with NW + CRC patients. *ZAG* mRNA levels were negatively associated with body mass index (BMI) in sWAT but positively correlated with BMI in vWAT. *TNF-*α mRNA levels in vWAT of OB + CRC patients were significantly increased by 2.8-fold when compared with NW + CRC patients. In particular, CRC was independently associated with serum ZAG levels. The risk of CRC in participants with high tertile serum ZAG levels was 5.84-fold higher than in those with low tertile ZAG levels after adjusting for age, gender, and other confounders [odds ratio (OR) = 6.84, 95% confidence interval (CI) 1.70–27.54, *P* = 0.03]. The CRC risk in participants with high tertile leptin levels was only 10.7% of those with low tertile leptin levels (OR = 0.11, 95% CI 0.01–0.89, *P* = 0.04). The area under the receiver operating characteristic (ROC) curve of ZAG was 0.66 (95% CI 0.54–0.77, *P* < 0.05). At the cutoff value of 1.42 µg/mL serum ZAG, the sensitivity and specificity for differentiating patients with CRC from controls were 62.2 and 69.2%, respectively.

**Conclusion:**

Serum ZAG levels were significantly increased in CRC patients. Subjects with higher circulating ZAG and lower leptin levels were more likely to have CRC than those with lower ZAG and higher leptin levels. Serum ZAG might be a potential diagnostic biomarker for CRC in the Chinese population.

## Introduction

Colorectal cancer (CRC) is the third most predominant cancer in men and the second in women around the world (1). Approximately one million new cases of CRC are diagnosed every year, and half a million people die yearly from this cancer worldwide (2). Though the etiology and pathogenesis of CRC is still unclear, a growing body of evidence has shown that obesity, particularly visceral obesity, is a risk factor for CRC (3, 4). It has been reported that the risk of CRC increases by 7 and 4%, respectively, for every 2 kg/m^2^ increase in body mass index (BMI) or 2 cm increase in waist circumference (4). Furthermore, increasing adiposity may influence its prognosis, including the recurrence, disease-free survival, and mortality of patients with CRC (5).

Although the mechanisms by which obesity contributes to the occurrence and development of CRC are multifactorial and have not yet been fully elucidated, accumulating evidence has shown that adipose tissue dysfunction in obesity, which causes an alteration of adipokine secretion, may mediate the relationship between obesity and CRC (6–8). Among these adipokines, adiponectin, leptin, and tumor necrosis factor-alpha (TNF-α) have been largely reported to be implicated in the development of CRC. Recent studies reported a significant inverse association of total and high-molecular-weight adiponectin (HMW-ADPN) with colorectal adenoma (9), not only for early CRC but also for advanced CRC patients (10). Studies in Western populations performed by Kumor and Salageanu et al. observed significantly lower serum leptin levels in CRC patients than in controls (11, 12). Additionally, a case-control study performed by Joshi et al. in a South Korean population found a negative association between leptin and CRC risk (13). TNF-α is usually considered to be a powerful anticancer agent because of its ability to induce necrosis of cancers. However, in recent years, accumulating evidence has demonstrated that TNF-α is increased during obesity (14) and may serve as a pro-cancer cytokine that is involved in carcinogenesis and cancer progression (15, 16). Higher serum levels of TNF-α have been shown to be associated with an increased risk of colorectal adenomas (17).

Zinc-α2-glycoprotein (ZAG, also called AZGP1) is a newly identified adipokine that is downregulated in obese patients and obese mice (18, 19). Recent studies have found that ZAG is also expressed in several malignancies, such as prostate, breast, and lung cancer (20–22), and the diagnostic value of serum ZAG in prostate cancer patients has also been reported (23). ZAG production is associated with the histological grade of prostate and breast cancer (24, 25). Thus, it is reasonable for us to wonder whether ZAG has any effect on CRC development and progression.

In the context of a role of ZAG in patients with CRC, so far, only three studies have been published (26–28). Early in 2012, Agesen et al. found high *ZAG* gene expression in the tumor tissue of CRC patients by using exon-level microarrays in a multi-medical center, multi-ethnic (Norwegian, USA, and Australia) and large-scale sample study (26). Ji et al. found the elevated ZAG levels in the sera and tumor tissues of CRC patients, and the elevated serum ZAG levels in CRC patients were correlated with an advanced clinical stage and poor prognosis (27). They also showed that the area under the curve (AUC) of the receiver operating characteristic (ROC) curve of ZAG was 0.95, which suggested that ZAG might be used as a potential serum biomarker for the diagnosis and prognosis of CRC patients (27). Studies by Xue et al. further suggested that the predictive diagnostic value of ZAG in serum was higher than carbohydrate 19-9 (CA19-9) but lower than carcinoembryonic antigen (CEA) (28). All these findings suggest that ZAG may play an important role in the development and progression of CRC. However, as we know, obesity alters the expression of ZAG (18, 19) and might affect the pathogenesis of CRC. Thus, studies on the role of ZAG in CRC should be undertaken separately in normal weight (NW) and overweight/obese (OW/OB) CRC patients. In addition, the previous studies mentioned above all focused on ZAG expression in normal and carcinoma tissues. Given that ZAG is an adipokine that can be secreted from adipose tissue, it is necessary to explore the expression of ZAG in subcutaneous and visceral white adipose tissue (sWAT and vWAT) in CRC patients.

Thus, the aim of our present study was (i) to provide serum ZAG profiles in three different groups (NW + CRC patients, OW/OB + CRC patients, and healthy controls); (ii) to investigate the mRNA expressions of *ZAG* in sWAT and vWAT in NW + CRC and OB + CRC patients; and (iii) to assess the association between circulating ZAG concentrations and the risk of CRC. In addition, three other adipokines—HMW-ADPN, leptin, and TNF-α—were also assessed in this study.

## Materials and Methods

### Study Subjects

A total of 76 CRC patients (38 with colon cancer and 38 with rectal cancer) who underwent surgery at the Department of General Surgery of Peking Union Medical College Hospital from June 2012 to April 2014 were recruited. All included participants were pathologically confirmed with colon/rectal cancer. BMI was calculated as weight (kilograms) divided by height (square meters). Patients with acute inflammatory disease, chronic rheumatic diseases, or other malignant tumors and those with BMI <18 kg/m^2^ were excluded from this study. In addition, 40 healthy subjects (18 kg/m^2^ < BMI < 25 kg/m^2^) were collected from the physical examination center with normal liver, kidney, and heart function and normal routine blood and urine tests, and their systolic blood pressure (SBP) and diastolic blood pressure (DBP) were also in the normal ranges. Informed consent was signed by all participants, and the study was approved by the ethics committee of Peking Union Medical College Hospital (No. S-364).

### Blood and Tissue Sample Collection and Processing

Colorectal cancer patients were divided into NW + CRC (18 kg/m^2^ < BMI < 25 kg/m^2^, *n* = 42) and OW/OB + CRC (BMI ≥ 25 kg/m^2^, *n* = 34) groups. All subjects had fasted overnight for at least 12 h, and blood samples were collected before the surgical operation. Serum was separated by centrifugation at 3,000 *g* for 10 min at 4°C and was stored in 1.5 mL Eppendorf tubes at −80°C for further analysis. In addition, sWAT and vWAT were obtained during the surgical procedure in nine OB + CRC patients and nine age-sex matched NW + CRC patients. Samples of adipose tissue were immediately frozen in liquid nitrogen and subsequently stored at −80°C for further study.

### Serum Biochemical Parameters and Adipokine Measurements

Serum total cholesterol (TC), triglycerides (TG), low-density lipoprotein cholesterol (LDL-C), high-density lipoprotein cholesterol (HDL-C), and fasting blood glucose (FBG) levels were determined by routine automated laboratory methods in our clinical laboratory. Serum adipokines including ZAG, HMW-ADPN, leptin, and TNF-α were measured by commercially available enzyme-linked immunosorbent assay kits (USCN Life Science Inc., Wuhan, China) according to the manufacturer’s instructions. The low limits of detection for ZAG, HMW-ADPN, leptin, and TNF-α were 1.80 ng/mL, 0.07 ng/mL, 0.06 ng/mL, and 6.50 pg/mL, respectively. The intra- and inter-assay coefficients of variation were 1.13 and 8.52% for ZAG, 0.59 and 4.60% for HMW-ADPN, 1.72 and 2.32% for leptin, and 2.07 and 2.76% for TNF-α.

### Total RNA Preparation and Reverse Transcription Quantitative PCR

Total RNA was extracted from human sWAT and vWAT by using E.Z.N.A Total RNA Kit I (Omega, San Diego, CA, USA) according to the manufacturer’s recommendations. Total RNA concentrations were estimated by Nano Drop 2000C (Thermo, Forma, USA). Then, 1.0 µg of total RNA was reverse transcribed into cDNA by using 1.0 µL Omniscript reverse transcriptase (Qiagen, Hilden, Germany), 10 U RNase inhibitor and an Oligo-dT primer (Promega, Madison, WI, USA) at 37°C for 60 min. PCR amplification was performed on an ABI 7500 PCR instrument (Applied Biosystems, CA, USA) with each gene in duplicate. The reaction conditions consisted of an initial denaturation step (10 min at 95°C) and a cycling step (denaturation for 15 s at 95°C and annealing and extending for 1 min at 60°C for 40 cycles). β-Actin was used for normalization, and all the primer sequences used were listed in Table S1 in Supplementary Material. The results are expressed as fold changes of Ct value relative to controls by using the 2^−ΔΔCt^ formula (29).

### Statistical Analysis

Data are shown as the mean ± SD or median with interquartile range. Normal distribution of the variables was evaluated using the Shapiro–Wilk *W* test. Comparison of variables between two groups was performed by either the independent sample *t*-test or Mann–Whitney *U* test according to the data distribution. Univariate and multivariate logistic regression analyses were used to estimate the odds ratio (OR) and 95% confidence intervals (CIs) of each variable for CRC. Cutoff point analysis, defined by the largest distance from the diagonal line of the ROC curve [sensitivity × (1 − specificity)], was used to identify the optimal value of serum ZAG levels that differentiated healthy people from patients with CRC. The sensitivity and specificity of the index for the cutoff point were also calculated. Stepwise multiple regression analysis was performed to explore the variables independently related to ZAG levels in serum and WAT. All statistical computations were run on SPSS 20.0 for Windows (SPSS Inc., Chicago, IL, USA). *P* < 0.05 was considered statistically significant.

## Results

### General Characteristics of Subjects in NW + CRC, OW/OB + CRC, and Control Groups

The characteristics of the CRC patients and healthy controls have been summarized in Table 1. Generally, NW + CRC patients have a significantly higher age but lower HDL-C levels when compared with control subjects (all *P* < 0.05). As expected, patients in the OW/OB + CRC group presented with a higher body weight, BMI, SBP, and TG than those in the NW + CRC group (all *P* < 0.05). However, no significant difference was observed with regard to height, DBP, FBG, TC, or LDL-C in these two groups.

**Table 1:** General characteristics of subjects in NW + CRC, OW/OB + CRC, and control groups.

Characteristics	Controls (*n* = 40)	Patients (*n* = 76)		
		All CRC (*n* = 76)	NW + CRC (*n* = 42)	OW/OB + CRC (*n* = 34)
Gender (M:F)	30/10	48/28	29/13	19/15
Age (years)	63.6 ± 7.4	67.5 ± 10.8^a^	67.9 ± 9.4^a^	67.1 ± 12.5
Height (cm)	166.5 ± 8.0	166.3 ± 7.8	166.5 ± 6.3	165.9 ± 9.3
Body weight (kg)	63.2 ± 7.8	69.3 ± 12.8^a^	61.2 ± 6.3	79.3 ± 11.7^b^
BMI (kg/m^2^)	22.5 ± 1.9	25.0 ± 3.9^a^	22.0 ± 1.6	28.7 ± 2.5^b^
Hypertension (%)	0	33 (43.4%)	17 (40.5%)	16 (47.1%)
Type 2 DM (%)	0	10 (13.2%)	4 (9.5%)	6 (17.6%)
Cardiovascular disease (%)	0	9 (11.8%)	4 (9.5%)	5 (14.7%)
SBP (mmHg)	121.3 ± 9.8	127.0 ± 14.6^a^	123.4 ± 15.4	131.5 ± 12.4^b^
DBP (mmHg)	74.7 ± 6.9	74.5 ± 9.8	73.5 ± 9.8	75.7 ± 9.7
FBG (mmol/L)	5.04 ± 0.43	5.65 ± 1.62^a^	5.45 ± 1.50	5.89 ± 1.75
TC (mmol/L)	4.79 ± 0.59	4.81 ± 0.97	4.62 ± 0.75	5.04 ± 1.16
TG (mmol/L)	1.23 ± 0.50	1.37 ± 0.57	1.21 ± 0.52	1.59 ± 0.56^b^
HDL-C (mmol/L)	1.37 ± 0.32	1.09 ± 0.23^a^	1.13 ± 0.26^a^	1.04 ± 0.18
LDL-C (mmol/L)	2.90 ± 0.48	2.95 ± 0.72	2.86 ± 0.67	3.07 ± 0.78

*Values are mean ± SD*.

*NW, normal weight; OW/OB, overweight/obese; CRC, colorectal cancer; BMI, body mass index; DM, diabetes mellitus; SBP, systolic blood pressure; DBP, diastolic blood pressure; FBG, fasting blood glucose; TC, total cholesterol; TG, triglycerides; HDL-C, high-density lipoprotein cholesterol; LDL-C, low-density lipoprotein cholesterol*.

*^a^P < 0.05 compared with control group*.

*^b^P < 0.05 compared with NW + CRC group*.

### Serum Levels of ZAG, Leptin, HMW-ADPN, and TNF-α in CRC Patients and Healthy Controls

**Figure 1:**
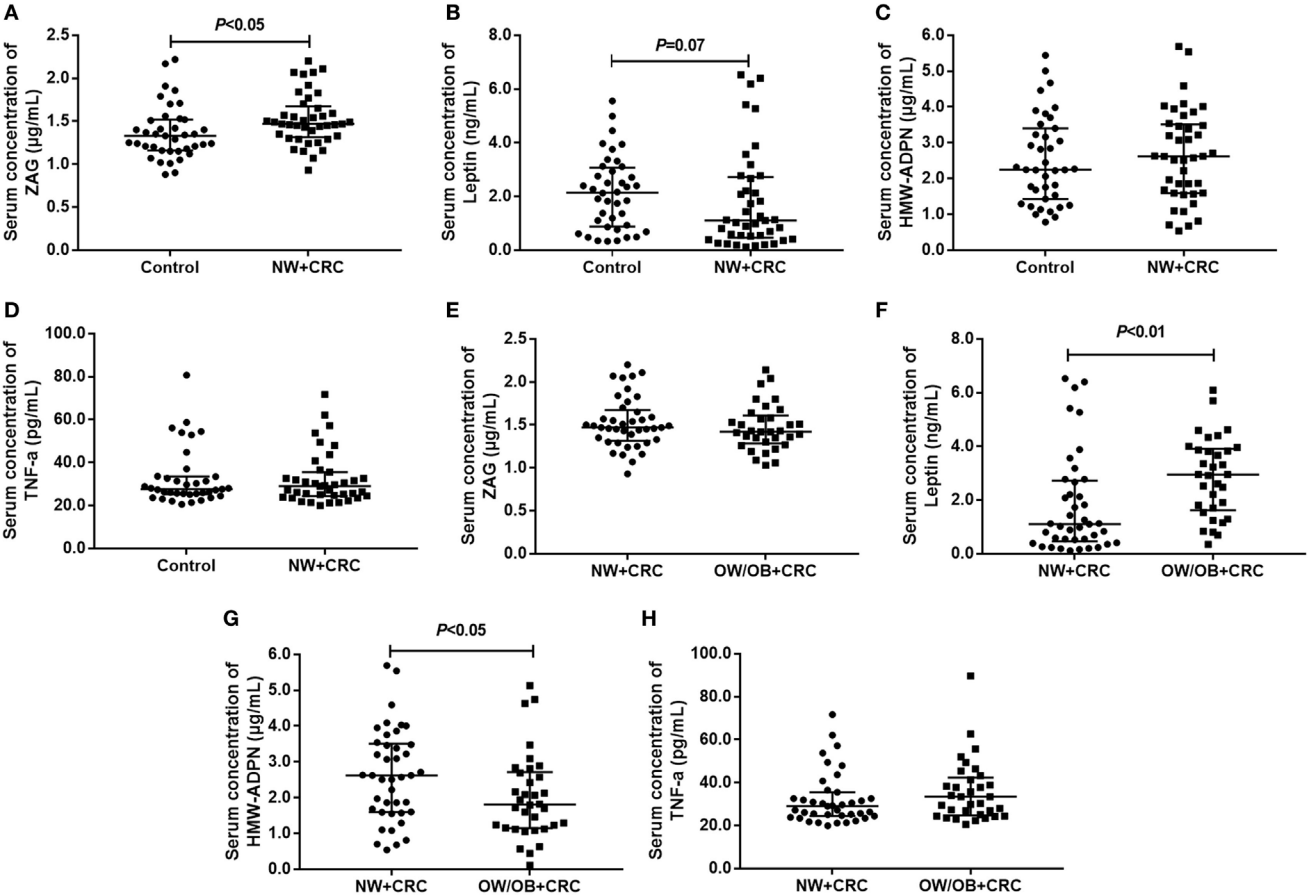
Serum levels of ZAG, leptin, HMW-ADPN, and TNF-α in NW + CRC patients and healthy controls **(A–D)** as well as in NW + CRC and OW/OB + CRC patients **(E–H)**. Abbreviations: ZAG, zinc-α2-glycoprotein; HMW-ADPN, high-molecular-weight- adiponectin; TNF-α, tumor necrosis factor-alpha; NW, normal weight; OW/OB, overweight/obese; CRC, colorectal cancer. All values are expressed as the median with the interquartile range.

As shown in Figure 1A, serum ZAG levels in NW + CRC patients were 11.7% higher than in healthy controls (1.53 ± 0.30 vs. 1.37 ± 0.31 μg/mL, *P* < 0.05). In addition, serum levels of leptin in NW + CRC patients had a tendency to be lower compared with healthy controls (1.82 ± 1.85 vs. 2.13 ± 1.36 ng/mL, *P* = 0.07) (Figure 1B). No significant difference was found in serum HMW-ADPN and TNF-α levels between NW + CRC patients and healthy controls (Figures 1C,D).

As shown in Figures 1E–H, serum HMW-ADPN levels in OW/OB + CRC patients were significantly decreased by 23.5% (2.02 ± 1.19 vs. 2.64 ± 1.30 μg/mL, *P* < 0.05), while the leptin levels were significantly increased by 57.7% (2.87 ± 1.47 vs. 1.82 ± 1.90 ng/mL, *P* < 0.01) when compared with NW + CRC patients. However, no significant difference was observed in serum ZAG and TNF-α levels between these two groups.

Next, serum levels of the four adipokines were further analyzed in male and female subjects, separately. As shown in Figure S1 in Supplementary Material, for men, serum ZAG levels in NW + CRC patients were 22.2% higher (1.57 ± 0.31 vs. 1.28 ± 0.25 μg/mL, *P* < 0.05), while the leptin levels were 30.1% lower (1.20 ± 1.36 vs. 1.71 ± 1.10 ng/mL, *P* < 0.05) than in healthy controls. Serum HMW-ADPN levels in male OW/OB + CRC patients were significantly decreased by 37.5% (1.67 ± 1.05 vs. 2.68 ± 1.41 μg/mL, *P* < 0.05), while the leptin levels were significantly increased by 86.1% (2.22 ± 1.29 vs. 1.20 ± 1.36 ng/mL, *P* < 0.01) when compared with NW + CRC patients. However, no significant difference was observed in serum ZAG, leptin, HMW-ADPN, and TNF-α levels in women across these three groups (Figure S2 in Supplementary Material).

### Expression of *ZAG, Leptin, HMW-ADPN*, and *TNF-*α in sWAT and vWAT of NW + CRC and OB + CRC Patients

**Figure 2:**
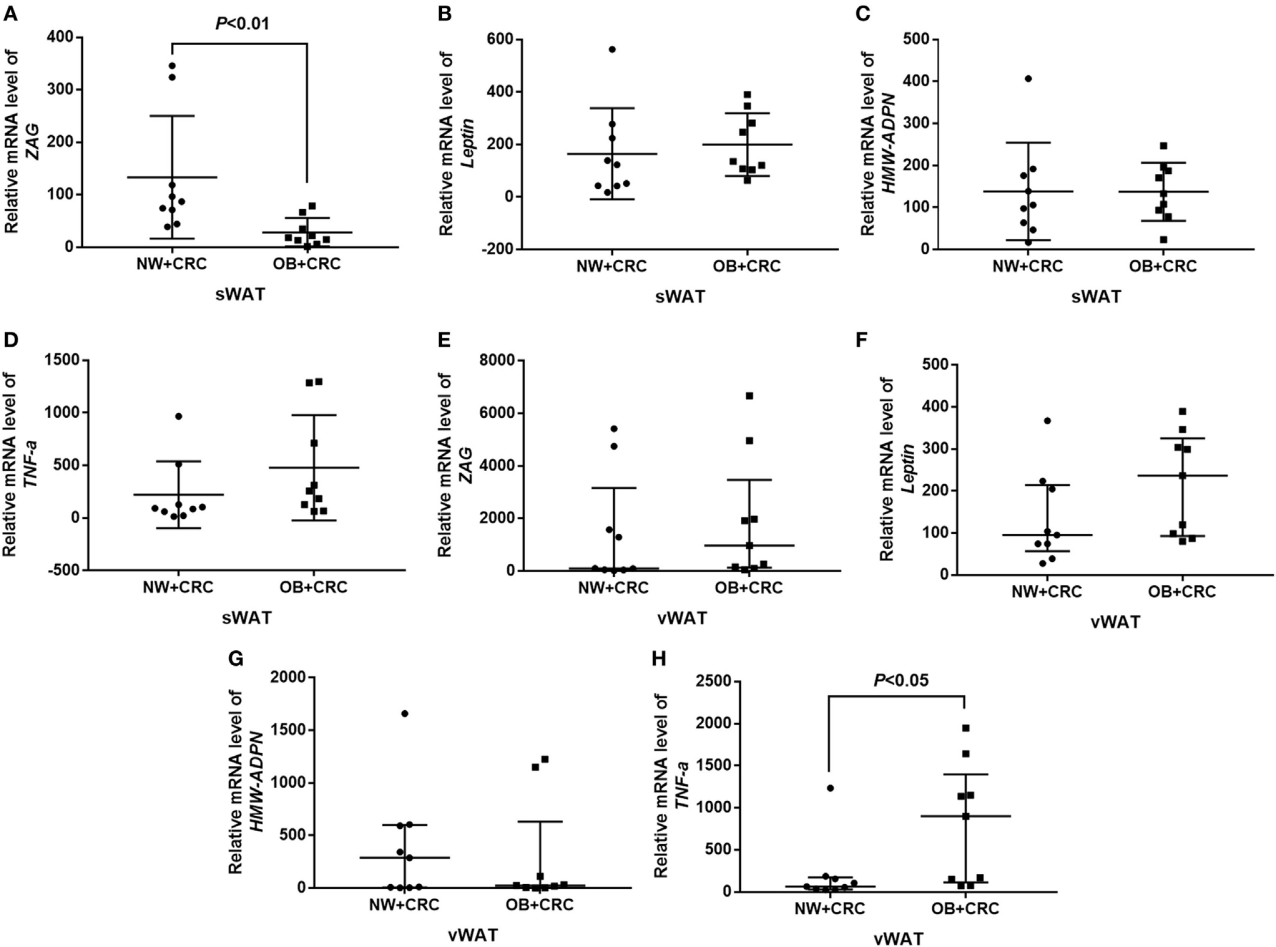
ZAG, leptin, HMW-ADPN, and TNF-α mRNA levels in sWAT **(A–D)** and vWAT **(E–H)** of NW + CRC and OB + CRC patients. Abbreviations: ZAG, zinc-α2-glycoprotein; HMW-ADPN, high-molecular-weight-adiponectin; TNF-α, tumor necrosis factor-alpha; sWAT, subcutaneous white adipose tissue; vWAT, visceral white adipose tissue; NW, normal weight; OB, obese; CRC, colorectal cancer. All values are expressed as the median with the interquartile range.

In our present study, the mRNA levels of *ZAG, leptin, HMW-ADPN*, and *TNF-*α were also measured in sWAT and vWAT from nine NW + CRC and nine OB + CRC patients. Baseline characteristics of the patients have been summarized in Table S2 in Supplementary Material. Our results showed that *ZAG* mRNA levels in sWAT were significantly lower in OB + CRC patients than in NW + CRC patients (reduced by 78.8%, *P* < 0.01) as presented in Figure 2A. Additionally, *TNF-*α mRNA levels in vWAT of OB + CRC patients were significantly increased by 2.8-fold when compared with NW + CRC patients (*P* < 0.05) (Figure 2H). No significant differences in *HMW-ADPN* or *leptin* mRNA levels in sWAT and vWAT were observed between these two groups (Figures 2B–G).

### The Relationships Between Clinical Parameters and Serum ZAG Levels and *ZAG* mRNA Levels in sWAT and vWAT in CRC Patients

As shown in Table 2, a significant positive correlation between circulating ZAG levels and TNF-α levels was found after adjusting for age and sex in CRC patients (*r* = 0.27, *P* = 0.03). *ZAG* mRNA levels in sWAT were negatively associated with BMI (*r* = −0.55, *P* = 0.03), whereas *ZAG* mRNA levels in vWAT were positively correlated with BMI (*r* = 0.60, *P* = 0.01). In addition, *ZAG* mRNA levels in sWAT were also found to be positively associated with serum HMW-ADPN levels (*r* = 0.54, *P* = 0.03). No significant relationship between serum ZAG levels and BMI was found in CRC patients.

**Table 2:** Partial correlation analysis between ZAG levels in both serum and WAT and clinical parameters in CRC patients.

	Serum ZAG (*n* = 76)		sWAT ZAG (*n* = 18)		vWAT ZAG (*n* = 18)	
	*r*	*P*	*r*	*P*	*R*	*P*
Age (years)	−0.15	0.21	−0.03	0.93	−0.25	0.33
BMI (kg/m^2^)	−0.15	0.23	−0.55	**0.03**	0.60	**0.01**
SBP (mmHg)	−0.04	0.75	−0.32	0.25	−0.29	0.29
DBP (mmHg)	−0.04	0.77	−0.41	0.13	0.01	0.98
FBG (mmol/L)	0.04	0.77	0.04	0.88	0.03	0.91
TC (mmol/L)	−0.04	0.77	−0.21	0.46	0.26	0.35
TG (mmol/L)	−0.02	0.10	−0.08	0.80	−0.30	0.29
HDL-C (mmol/L)	0.13	0.30	−0.02	0.95	−0.09	0.74
LDL-C (mmol/L)	−0.16	0.17	−0.14	0.63	0.28	0.31
Serum ZAG (μg/mL)	–	–	−0.10	0.73	0.08	0.77
Serum leptin (ng/mL)	−0.02	0.84	−0.52	0.06	0.52	0.06
Serum HMW-ADPN (μg/mL)	0.01	0.97	0.54	**0.03**	−0.46	0.07
Serum TNF-α (pg/mL)	0.27	**0.03**	−0.34	0.24	−0.1	0.68

*Values are age-sex-adjusted Spearman partial correlation coefficients*.

BMI, body mass index; SBP, systolic blood pressure; DBP, diastolic blood pressure; FBG, fasting blood glucose; TC, total cholesterol; TG, triglycerides; HDL-C, high-density lipoprotein cholesterol; LDL-C, low-density lipoprotein cholesterol; ZAG, zinc-α2-glycoprotein; HMW-ADPN, high-molecular-weight-adiponectin; TNF-α, tumor necrosis factor-alpha; sWAT, subcutaneous white adipose tissue; vWAT, visceral white adipose tissue.

*Bold font means p < 0.05*.

Next, stepwise multivariate linear regression was performed. As displayed in Table 3, CRC, TNF-α, and HDL-C were independent factors associated with serum ZAG levels after adjusting for age, gender, BMI, TC, TG, SBP, DBP, FBG, HDL-C, and LDL-C. Among them, the presence of CRC was found to be independently positively associated with serum ZAG levels (*B* = 0.24, *P* < 0.01), which was consistent with the higher serum ZAG levels in CRC patients as displayed in Figure 1A. Serum TNF-α levels were also independently positively associated with serum ZAG levels (*B* = 0.01, *P* < 0.01), which was also in accordance with the results shown in Table 2 by partial correlation analysis. In addition, BMI was independently negatively related to *ZAG* mRNA levels in sWAT but independently positively related to *ZAG* mRNA levels in vWAT, which was also consistent with the results demonstrated in Table 2.

**Table 3:** Multiple regression analysis for the variables independently related to serum ZAG and *ZAG* mRNA in sWAT and vWAT in CRC patients.

	*B*	SE of *B*	β	*P*
**Serum ZAG (*R*^2^ = 0.52)**				
CRC (0 or 1)	0.24	0.06	0.34	<0.01
TNF-α	0.01	0.00	0.34	<0.01
HDL-C	0.31	0.10	0.31	<0.01
Constant	0.66	0.15		<0.01

***ZAG* mRNA in sWAT (*R*^2^ = 0.298)**				
BMI	−10.00	4.10	−0.55	0.03
Constant	351.44	112.27		0.01

***ZAG* mRNA in vWAT (*R*^2^ = 0.553)**				
BMI	243.68	66.76	0.70	<0.01
SBP	−83.08	33.38	−0.48	0.03
Constant	4,763.52	4,120.78		0.27

*Stepwise multiple regression analysis was used. Variables also entered into multiple regression analysis but not included in the equation: age, gender, BMI, total cholesterol, triglycerides, SBP, diastolic blood pressure, fasting blood glucose, low-density lipoprotein cholesterol, serum high-molecular-weight-adiponectin, and serum leptin*.

*B, regression coefficient; β, standardized regression coefficient; sWAT, subcutaneous white adipose tissue; vWAT, visceral white adipose tissue; 0; non-cancer; 1; colorectal cancer; ZAG, zinc-α2-glycoprotein; CRC, colorectal cancer; HDL-C, high-density lipoprotein cholesterol; BMI, body mass index; SBP, systolic blood pressure*.

### Association of ZAG, Leptin, HMW-ADPN, and TNF-α with CRC Risks

Next, all subjects were stratified into trisections according to ZAG tertiles (lowest: <1.30 μg/mL; median: 1.30–1.51 µg/mL; highest: ≥1.51 μg/mL). As shown in Table 4, the CRC risk was 2.43-fold higher in subjects with the high ZAG level than those with the low serum ZAG levels (OR = 3.43, 95% CI 1.24–9.49, *P* = 0.02) after adjusting for age and gender (Model 1). This increased probability of CRC risk still remained after further adjusting for BMI, SBP, DBP, and FBG based on Model 1 (Model 2, OR = 3.96, 95% CI 1.28–12.27, *P* = 0.04) and TC, TG, HDL-C, and LDL-C based on Model 2 (Model 3, OR = 6.84, 95% CI 1.70–27.54, *P* = 0.03). In addition, serum leptin levels were also categorized into tertiles (lowest: <1.12 ng/mL; median: 1.12–2.92 ng/mL; highest: ≥2.92 ng/mL). As presented in Table 4, the probability of CRC risk decreased by 94.0% in participants with the highest leptin level compared with those with the lowest serum leptin levels (OR = 0.06, 95% CI 0.01–0.41, *P* = 0.01) after adjusting for age, gender, BMI, SBP, DBP, and FBG (Model 2). This decreased risk of CRC remained after further adjusting for TC, TG, HDL-C, and LDL-C based on Model 2 (Model 3, OR = 0.11, 95% CI 0.01–0.89, *P* = 0.04). However, no significant differences were found in the OR of CRC risks between the tertiles of HMW-ADPN and TNF-α levels.

**Table 4:** Unconditional logistic regression analysis of colorectal cancer risks according to tertiles of zinc-α2-glycoprotein (ZAG), leptin, high-molecular-weight adiponectin (HMW-ADPN), and tumor necrosis factor-alpha (TNF-α) in all subjects.

	Tertile (number of cases and controls)			

Measurement	Lowest odds ratio (OR) [95% confidence interval (CI)]	Median OR (95% CI)	Highest OR (95% CI)	*P* for trend
**ZAG**				
Range (μg/mL)	<1.30	≥1.30 to <1.51	≥1.51	
Cases/controls	18/20	29/9	28/11	
Univariate	1.00 (reference)	3.06 (1.17–8.04)	2.85 (1.08–7.52)	**0.04**
Model 1	1.00 (reference)	3.35 (1.23–9.12)	3.43 (1.24–9.49)	**0.02**
Model 2	1.00 (reference)	2.97 (0.98–9.04)	3.96 (1.28–12.27)	**0.04**
Model 3	1.00 (reference)	2.52 (0.70–9.09)	6.84 (1.70–27.54)	**0.03**

**Leptin**				
Range (ng/mL)	<1.12	≥1.12 to <2.92	≥2.92	
Cases/controls	27/12	22/17	26/11	
Univariate	1.00 (reference)	0.65 (0.26–1.65)	1.14 (0.42–3.04)	0.46
Model 1	1.00 (reference)	0.60 (0.23–1.57)	0.76 (0.24–2.38)	0.58
Model 2	1.00 (reference)	0.13 (0.03–0.53)	0.06 (0.01–0.41)	**0.01**
Model 3	1.00 (reference)	0.13 (0.03–0.64)	0.11 (0.01–0.89)	**0.04**

**HMW-ADPN**				
Range (μg/mL)	<1.68	≥1.68 to <2.89	≥2.89	
Cases/controls	26/12	25/13	24/15	
Univariate	1.00 (reference)	0.79 (0.30–2.05)	0.70 (0.26–1.83)	0.76
Model 1	1.00 (reference)	0.61 (0.22–1.69)	0.56 (0.20–1.54)	0.49
Model 2	1.00 (reference)	0.95 (0.30–2.94)	1.17 (0.36–3.81)	0.92
Model 3	1.00 (reference)	0.92 (0.26–3.33)	1.59 (0.41–6.18)	0.65

**TNF-α**				
Range (pg/mL)	<25.93	≥25.93 to <33.43	≥32.43	
Cases/controls	26/12	21/17	28/11	
Univariate	1.00 (reference)	0.70 (0.27–1.83)	1.35 (0.50–3.68)	0.42
Model 1	1.00 (reference)	0.75 (0.27–2.05)	1.65 (0.58–4.71)	0.32
Model 2	1.00 (reference)	1.16 (0.36–3.80)	1.33 (0.40–4.44)	0.90
Model 3	1.00 (reference)	1.25 (0.31–4.97)	1.210 (0.32–4.54)	0.94

*Multivariate ORs and 95% CIs from unconditional logistic regression models were used in the analysis*.

*Model 1: basic model, adjusted for age and gender*.

*Model 2: further adjusted for body mass index, systolic blood pressure, diastolic blood pressure, and fasting blood glucose based on the model 1*.

Model 3: full model, further adjusted for total cholesterol (<5.18 mmol/L, ≥5.18 mmol/L), triglycerides (<1.7 mmol/L, ≥1.7 mmol/L), high-density lipoprotein cholesterol (<1.04 mmol/L, ≥1.04 mmol/L), and low-density lipoprotein cholesterol (<3.37 mmol/L, ≥3.37 mmol/L) based on the model 2.

*Bold font means p < 0.05*.

### Diagnostic Values of Serum ZAG, Leptin, HMW-ADPN, and TNF-α for CRC

**Figure 3:**
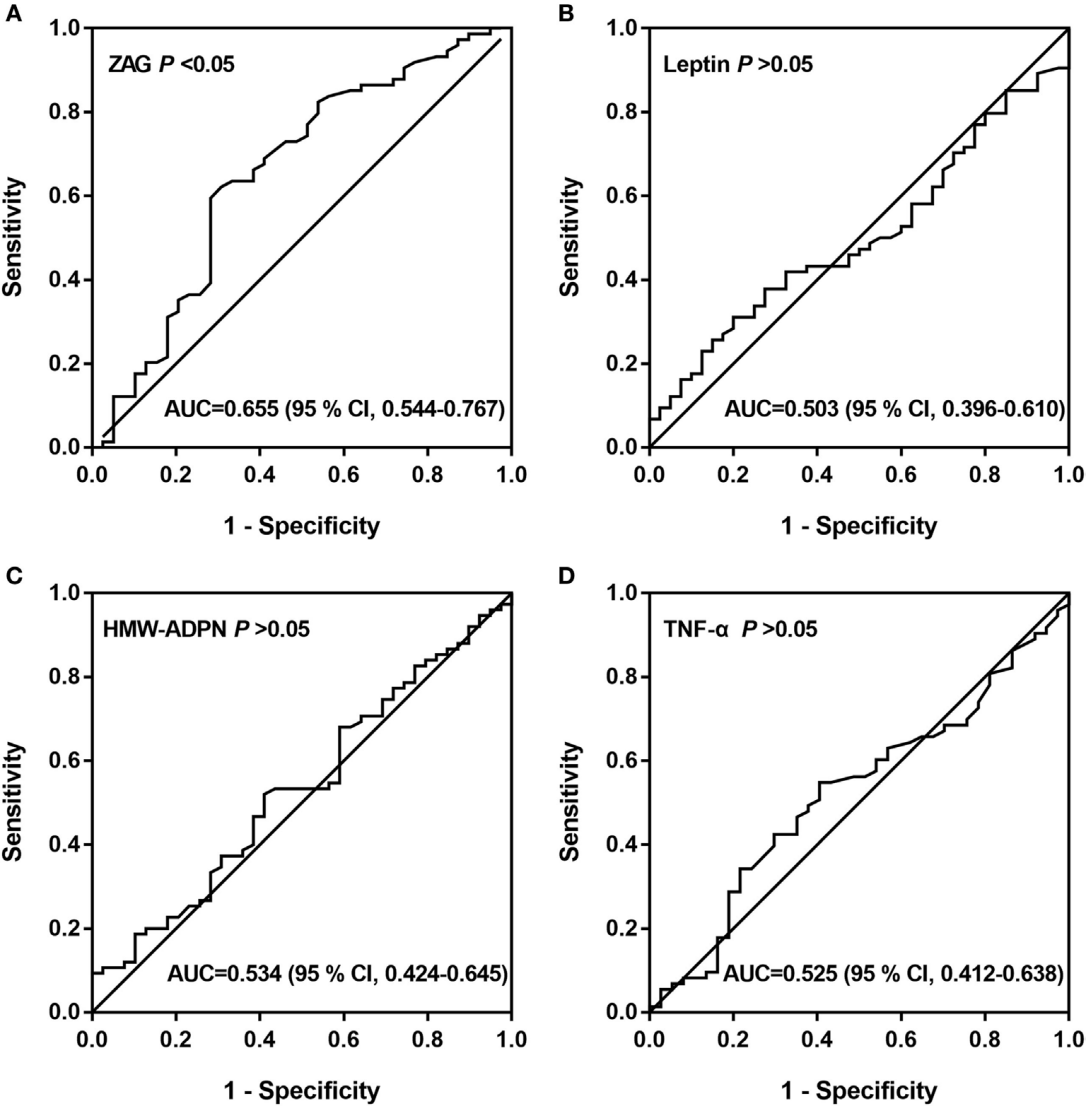
ROC curves of serum ZAG **(A)**, leptin **(B)**, HMW-ADPN **(C)**, and TNF-α **(D)**. ROC curves were derived by plotting the relationship between the specificity and the sensitivity at various cutoff levels. Abbreviations: ZAG, zinc-α2-glycoprotein; HMW-ADPN, high-molecular-weight-adiponectin; TNF-α, tumor necrosis factor-alpha; ROC, receiver operating characteristic; AUC, area under the curve.

Subsequently, ROC curve analysis was used to investigate the potential application of the four serum adipokines for the discrimination between patients with CRC and healthy people. Our results indicate that only ZAG can effectively discriminate between CRC patients and healthy individuals within ROC curve areas of 0.655 (95% CI 0.544–0.767, *P* < 0.05) (Figure 3A–D). At the cutoff value of 1.42 µg/mL for ZAG, the sensitivity and specificity for the discrimination of CRC are 62.2 and 69.2%, respectively.

## Discussion

Zinc-α2-glycoprotein is a 41-kDa secreted glycoprotein that was first identified in human plasma in 1961 (30). Previous studies have shown that ZAG is expressed at high levels in a variety of malignancies, such as prostate, breast, and lung cancer (20–22). Serum ZAG was found to be a potential biomarker for prostate cancer (23). Early in 2012, Agesen et al. found increased *ZAG* gene expression at the transcriptional levels in CRC tissues from Western populations, including Norwegian, American, and Australian populations (26). Further studies in Chinese populations also reported that ZAG was upregulated at the transcriptional and posttranscriptional levels in fresh colon cancer tissues (27), suggesting that ZAG might be a potential biomarker for CRC in both Western and Eastern populations. Consistent with its elevated expression in cancer tissues, Xue et al. also found higher serum ZAG levels in CRC patients than in healthy controls (28). Further analysis showed that there was a positive association between serum ZAG levels and CRC clinical stages (28). Another study performed by Ji et al. found that serum ZAG was elevated in CRC patients, and CRC patients with higher ZAG levels showed worse clinical outcomes (27). In our present study, we also found that serum ZAG levels were significantly increased in CRC patients with NW and CRC was found to be independently associated with serum ZAG levels. Based on these data, it is thus reasonable to assume that the elevated expression of ZAG in CRC tissues results in high serum ZAG levels, which may further promote CRC development. In addition, our study showed that the significantly higher serum ZAG levels only presented in male CRC patients, which is consistent with the epidemiological findings that men are at a higher risk of CRC.

Next, all subjects were stratified into trisections according to their serum ZAG tertiles. The results showed that the CRC risk was 2.43-fold higher in subjects with the highest ZAG level than those with the lowest serum ZAG levels after adjusting for age and gender (Model 1). This increased probability of CRC remained after further adjusting for BMI, SBP, DBP, and FBG based on Model 1 (Model 2, OR = 3.96) and TC, TG, HDL-C, and LDL-C based on Model 2 (Model 3, OR = 6.84), suggesting that ZAG overexpression is a significant risk factor for CRC, independent of other clinical pathological factors. Further analyses using ROC curves showed that the AUC of ZAG was 0.655. At the cutoff value of 1.42 µg/mL, the diagnostic value of ZAG had 62.2% sensitivity and 69.2% specificity. In accordance with our results, Ji et al. reported that serum ZAG was a useful biomarker for CRC within ROC curve areas of 0.9572 (95% CI 0.9173–0.9971) in a cohort of 534 Chinese individuals (27). Studies conducted by Xue et al. further found that the AUC of ZAG was 0.742 (95% CI 0.656–0.827), which was lower than the AUC of CEA (0.746, 95% CI 0.665–0.827) but higher than the AUC of CA19-9 (0.676, 95% CI 0.578–0.774) in a total of 160 Chinese subjects (28). All of these findings suggest that ZAG could be used as a potential serum biomarker for CRC.

Zinc-α2-glycoprotein is also a novel adipokine that can be secreted by adipose tissue. Decreased ZAG levels in sWAT of obese patients and its negative association with BMI have been previously reported (19, 31–33). In our present study, we observed for the first time that *ZAG* mRNA levels in sWAT of OB + CRC patients were also significantly decreased when compared with NW + CRC patients. Further partial correlation and multiple regression analysis found a negative relationship between *ZAG* mRNA levels in sWAT and BMI, and BMI was independently negatively related to *ZAG* levels in sWAT. These results suggest that the decreased ZAG levels in sWAT and its negative relationship with BMI were observed both in simple OW/OB patients and in CRC patients. By contrast, *ZAG* mRNA levels in vWAT were found to be positively related with BMI in our present study. In contrast with our findings in CRC patients, previous studies in simple obese patients performed by Mracek and Selva et al. found that ZAG expression in vWAT was significantly lower in obese patients and showed a negative correlation with BMI (19, 34). Given these results together, we speculate that *ZAG* mRNA levels in sWAT and vWAT might play a different role in CRC patients. Previous studies by Balaz have shown that ZAG in sWAT, but not in vWAT, is associated with whole-body insulin sensitivity (31). Although the biological mechanisms of the different role of sWAT and vWAT in CRC are still not well known, a possible explanation for this difference may be attributed to the much more severe insulin resistance of vWAT compared to sWAT (31). In addition, *ZAG* mRNA levels in vWAT were for the first time found to be negatively correlated with SBP in our present study. Further studies need to be done to validate this phenomenon.

After stratifying all subjects into trisections according to serum leptin tertiles, the probability of CRC risk was found to decrease by 94.0% in subjects with the highest leptin level compared to those with the lowest serum leptin levels after adjusting for age, gender, BMI, SBP, DBP, and FBG (Model 2, OR = 0.06). This decreased risk of CRC remained even after further adjusting for TC, TG, HDL-C, and LDL-C based on Model 2 (Model 3, OR = 0.11), suggesting that leptin might be a protective factor against CRC, which is independent of other clinical pathological factors. In line with our results, a case–control study performed by Joshi et al. in a South Korean population found a negative association between leptin and CRC risk (13). Additionally, studies in Western populations performed by Kumor and Salageanu et al. observed significantly lower serum leptin levels in CRC patients than controls (11, 12). Our present study also demonstrated that serum leptin levels have a lower trend in NW + CRC patients than healthy controls (*P* = 0.07).

Next, the CRC patients were further divided into OW/OB or NW groups. Interestingly, a significantly higher serum leptin levels was observed in OW/OB Chinese CRC patients when compared with NW CRC patients. Consistent with our results, studies performed by Stachowicz et al. in a total of 146 Caucasians with CRC also demonstrated that OW/OB CRC patients had statistically higher serum leptin levels than NW patients (35). It is well known that obese patients have markedly increased circulating leptin levels compared with NW controls (36). In our present study, we first found that the OW/OB CRC Chinese patients also had higher serum leptin than NW CRC patients.

ADPN is a 30-kDa protein hormone secreted exclusively from adipose tissue (37), and HMW-ADPN is now considered the most active form of adiponectin (38). ADPN has been shown to be decreased in obese subjects and is supposed to exert anti-inflammatory and anticancerous activity (39). Our present study also observed significantly decreased serum levels of HMW-ADPN in OW/OB + CRC patients compared to NW + CRC patients, similar to its profile in obese patients (40).

Tumor necrosis factor-alpha is a key pro-inflammatory cytokine produced by macrophages cells and secreted by adipocytes (41). It has been widely accepted that an obesity-associated low grade of chronic inflammation is an important contributing factor in CRC pathogenesis (42). However, in this study, no significant differences were found in the serum TNF-α level between NW + CRC patients and healthy controls, or between NW + CRC and OW/OB + CRC patients. In accordance with our results, Amor et al. measured the plasma levels of TNF-α in lean and obese subjects with and without CRC also found no significant change in plasma TNF-α levels between patients with CRC or obesity (43). Additionally, our study observed that *TNF-*α mRNA levels were upregulated in vWAT from OB + CRC patients compared to NW + CRC patients. Studies performed by Delgado et al. also reported that vWAT, but not sWAT, was an indicator of inflammation (44). These results suggest that TNF-α secreted by vWAT, instead of sWAT, may be involved in obesity-related CRC development.

In conclusion, our study found that serum ZAG levels were significantly increased in CRC patients. *ZAG* mRNA levels in sWAT were found to be significantly reduced in OB + CRC Chinese patients in comparison with NW + CRC patients. The patients with the highest tertile ZAG serum levels were more likely to have CRC. At the cutoff value of 1.42 µg/mL for serum ZAG, the sensitivity and specificity for differentiating patients with CRC from controls were 62.2 and 69.2%, respectively. Additional and more comprehensive studies are needed to explore the detailed mechanisms of the role of ZAG in CRC development.

## Ethics Statement

Informed consent was signed by all participants and the study was approved by the ethics committee of Peking Union Medical College Hospital (No. S-364).

## Author Contributions

HZ designed the experiments and revised the primary manuscript. ML analyzed the data and wrote the primary manuscript. NZ performed the molecular biological experiments. HP, GL, NL, LW, HY, and KY collected the clinical materials and serum samples and finished the clinical and biochemical parameters measurements. FG designed the experiments, supervised the whole study, and revised the primary manuscript.

## Conflict of Interest Statement

The authors declare that the research was conducted in the absence of any commercial or financial relationships that could be construed as a potential conflict of interest.

## Abbreviations

CRC, colorectal cancer; sWAT, subcutaneous white adipose tissue; vWAT, visceral white adipose tissue; NW, normal weight; OW/OB, overweight/obese; ZAG, zinc-α2-glycoprotein; HMW-ADPN, high-molecular-weight adiponectin; TNF-α, tumor necrosis factor-alpha; ELISA, enzyme-linked immunosorbent assay; ROC, receiver operating characteristic; BMI, body mass index; AUC, area under the curve; CA19-9, carbohydrate 19-9; CEA, carcinoembryonic antigen; SBP, systolic blood pressure; DBP, diastolic blood pressure; TC, total cholesterol; TG, triglycerides; LDL-C, low-density lipoprotein cholesterol; HDL-C, high-density lipoprotein cholesterol; FBG, fasting blood glucose; RT-qPCR, reverse transcription quantitative PCR; SD, standard deviation; OR, odds ratio; CI, confidence interval.
