# Supplementary material for: Circulating and Adipose Tissue mRNA Levels of Zinc-α2-Glycoprotein, Leptin, High-Molecular-Weight Adiponectin, and Tumor Necrosis Factor-Alpha in Colorectal Cancer Patients With or Without Obesity
Source: Front Endocrinol (Lausanne). 2018 Apr 26;9:190. doi: 10.3389/fendo.2018.00190 (PMC5932179; doi:10.3389/fendo.2018.00190)
Supplement: Supplementary file 1 [file table_1.docx]

| **Table S1. Primers sequences used for qRT-PCR in human sWAT and vWAT** | | |
| --- | --- | --- |
| **Gene** | **Forward primer** | **Reverse primer** |
| *β-actin* | TCCCTGGAGAAGAGCTACG | GTAGTTTCGTGGATGCCACA |
| *ZAG* | GCTTACCTGGAGGAGGAGTG | TTCCCTGGGTAGAAGTCGTAG |
| *Leptin* | TTTCACACACGCAGTCAGTCTC | CTGGAAGGCATACTGGTGAGGAT |
| *ADPN* | AGGCCGTGATGGCAGAGAT | TCCAATCCCACACTGAATGCT |
| *TNF-α* | ACCACGCTCTTCTGCCTGCT | GGGTTTGCTACAACATGGGCTACA |

Abbreviations: ZAG, zinc-α2-glycoprotein; ADPN, adiponectin; TNF-α, tumor necrosis factor-alpha; sWAT, subcutaneous white adipose tissue; vWAT, visceral white adipose tissue.
