# Supplementary figures and images for: Circulating and Adipose Tissue mRNA Levels of Zinc-α2-Glycoprotein, Leptin, High-Molecular-Weight Adiponectin, and Tumor Necrosis Factor-Alpha in Colorectal Cancer Patients With or Without Obesity
Source: Front Endocrinol (Lausanne). 2018 Apr 26;9:190. doi: 10.3389/fendo.2018.00190 (PMC5932179; doi:10.3389/fendo.2018.00190)

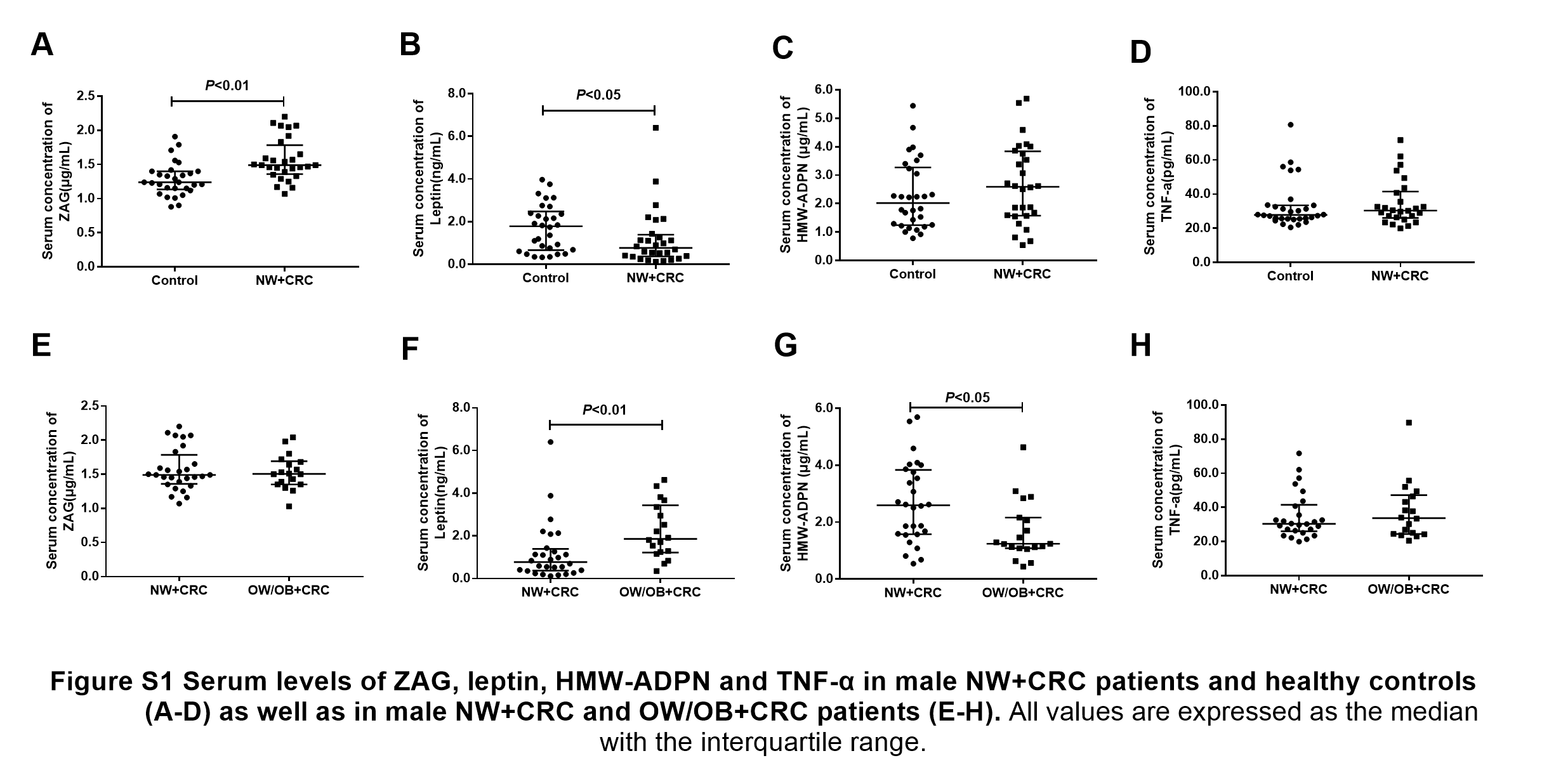

Supplement: Supplementary file 3 [file image_1.tif]

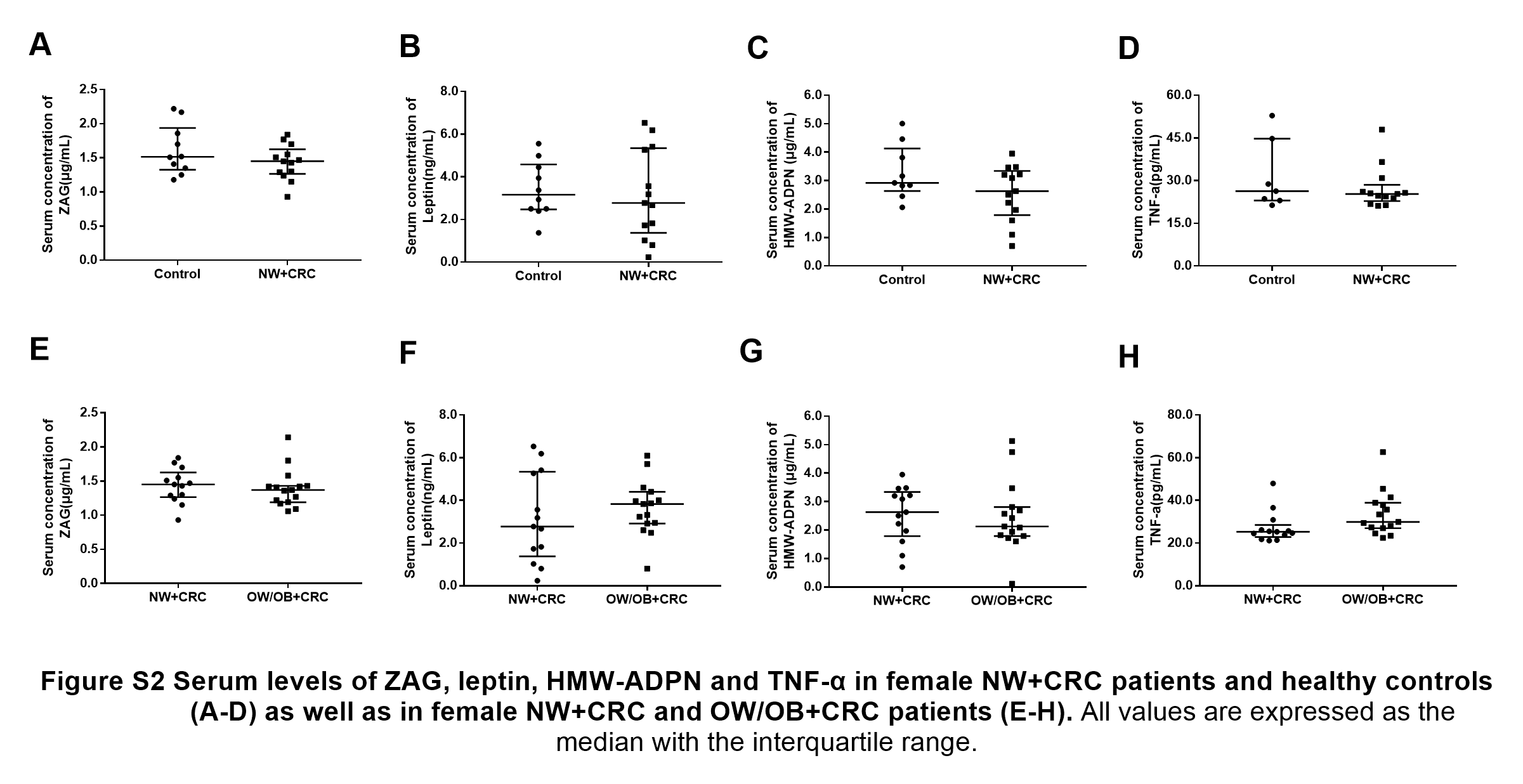

Supplement: Supplementary file 4 [file image_2.tif]
